# The impact of team cohesion on athlete engagement in collegiate basketball leagues: the moderating role of paternalistic leadership

**DOI:** 10.3389/fpsyg.2024.1473506

**Published:** 2024-12-10

**Authors:** Liang Wang, Jianhua Xu, Yao Liu

**Affiliations:** School of Physical Education and Sport Science, Fujian Normal University, Fuzhou, Fujian, China

**Keywords:** team cohesion, athlete engagement, paternalistic leadership, moderating effects, college basketball players

## Abstract

**Objective:**

This study investigates the impact of team cohesion on the engagement of college basketball players, and examines the moderating effects of authoritarian, benevolent, and moral leadership styles within paternalistic leadership on this influence. This provides theoretical insights for improving athlete engagement.

**Methods:**

Data from 404 athletes participating in the 26th China Collegiate Basketball League were collected through a questionnaire survey and analyzed statistically.

**Results:**

(1) Team cohesion has a significant positive impact on athlete engagement in collegiate basketball players. (2) Authoritarian leadership has a significant negative moderating effect on the relationship between team cohesion and athlete engagement in collegiate basketball players; the stronger the authoritarian leadership, the weaker the positive impact of team cohesion on athlete engagement in collegiate basketball players. This effect diminishes and becomes statistically insignificant when the authoritarian leadership score exceeds 5.88. (3) Benevolent leadership does not have a significant moderating effect between team cohesion and athlete engagement in collegiate basketball players. (4) Moral leadership has a significant positive moderating effect on the relationship between team cohesion and athlete engagement in collegiate basketball players; the higher the moral leadership, the stronger the positive impact of team cohesion on athlete engagement in collegiate basketball players, which becomes insignificant when the moral leadership score is below 2.75.

**Conclusion:**

Enhancing team cohesion is an effective method to increase athlete engagement in collegiate basketball players. It is advisable for collegiate basketball coaches to refrain from adopting controlling and demeaning authoritative approaches, and instead, emphasize ethical leadership practices, continually enhancing their own abilities and virtues to serve as role models.

## 1 Introduction

Under the significant pressures of long-term intensive training and competition, many elite athletes have exhibited psychological health issues such as fatigue and burnout, with some even choosing to end their sports careers prematurely. Mental health plays a crucial role in the careers of athletes, not only concerning personal well-being but also as a key factor in maintaining athletic achievements (Henriksen et al., 2020). Historically, research primarily focused on negative psychological issues like stress, fatigue, and burnout among athletes. However, with the development of positive psychology, the research perspective has shifted toward exploring human strengths and virtues, aiming to enhance athletes’ mental health from a positive standpoint. Engagement, as the positive counterbalance to burnout (Schaufeli et al., 2002), has increasingly captured scholars’ interest. Athlete engagement is characterized as a persistent, positive cognitive and emotional state within sports, predominantly marked by confidence, dedication, vigor, and enthusiasm (Lonsdale et al., 2007). Zhang (2012) has specifically identified athlete engagement as a critical indicator of the positive psychological aspects of athletes. It not only reflects their level of psychological health but also fosters optimism, resilience, and creativity. These qualities significantly influence athletes’ growth, competitive ability, and performance (Zhang, 2012). Subsequent empirical studies by scholars have demonstrated that athlete engagement not only mitigates sports-related psychological fatigue (Guo et al., 2021, 2022), but also significantly enhances satisfaction with sports performance (Ye et al., 2016) and overall sports success (Ma, 2021). Athletes in collegiate basketball leagues, who not only serve as a talent reservoir for the sport (Zhang, 2007) but also play a key role in the nationwide fitness program, are vital. Maintaining their participation in sports and achieving good results are crucial, and thus enhancing the level of athlete engagement is essential. How can we enhance the engagement levels of college basketball players?

Based on Social Information Processing Theory, it is understood that an individual’s environment can significantly influence their cognition and behavior (Li and Xu, 2019). Team cohesion plays a critical role in group development, maintenance, and goal achievement. It reflects athletes’ perceptions of the team atmosphere and is a crucial situational factor (Ma and Wang, 2006). Existing studies have shown that team cohesion significantly affects the physical and psychological well-being of athletes (Gu et al., 2022; Yildirim et al., 2024), and its influence is particularly notable in basketball and other team sports that require close cooperation among team members to achieve victory. The extensive interaction time between athletes during training and competitions further accentuates the impact of team cohesion. Therefore, what impact does team cohesion have on college basketball players?

College basketball players are at a crucial stage of rapid personality development and maturation. However, due to the similar ages and limited life experiences of the team members, they are particularly prone to psychological health issues during this period. In this context, the role of the coach becomes particularly critical. Coaches are responsible not only for technical guidance but also play a vital role in fostering the psychological health and growth of their players. The leadership style of coaches becomes an important organizational situational factor influencing team members’ behaviors and can affect them on multiple levels (Amabile et al., 2004). A coach’s leadership style can directly impact athletes and also serve as a moderating variable, adjusting the effects of other factors on athletes’ behavioral attitudes. However, most current research focuses on the direct effects of coaches’ leadership styles on athlete engagement or team cohesion (You, 2014; Gao et al., 2021; Leo et al., 2022; Xu, 2022), with insufficient attention given to the moderating role of different coaching styles. Paternalistic leadership, a leadership theory developed within Chinese culture, is prevalent among Chinese coaches (Lin et al., 2017). Recent studies have shown that this leadership style is also widely recognized in other Asian countries, such as Japan, South Korea, and Taiwan (Cheng et al., 2014). Moreover, the leadership approach of legendary Western coach John Wooden exhibits many traits similar to paternalistic leadership (Jenkins, 2014), demonstrating the cross-cultural applicability of this leadership style. Different coaches exhibit varying paternalistic leadership styles, and athletes’ perceptions of these styles can vary significantly. If coaches do not adopt an appropriate leadership style, team cohesion might not effectively positively influence athletes’ engagement and could even have negative effects. Therefore, clearly understanding the moderating role of paternalistic leadership between team cohesion and athlete engagement in collegiate basketball players, as well as its specific manifestations, is of significant importance for improving team performance and guiding the holistic development of athletes’ physical and mental health.

Accordingly, this study aims to explore: (1) the impact of team cohesion on athlete engagement in collegiate basketball players; (2) the specific moderating roles of the three types of paternalistic leadership within the relationship between team cohesion and athlete engagement in collegiate basketball players.

## 2 Theoretical review and research hypotheses

### 2.1 Team cohesion and athlete engagement

Team cohesion reflects the dynamic process by which a team unites and aligns itself while pursuing instrumental goals or satisfying members’ emotional needs (Potrac et al., 2013). It is considered one of the most significant social contexts influencing individual work behaviors (Zhao et al., 2021). According to Social Information Processing Theory, people’s attitudes and behaviors are largely influenced by their surrounding social environment, and the interpretation of this information determines subsequent attitudes and behaviors (Li and Xu, 2019). The team cohesion perceived by athletes naturally influences their behaviors. Moreover, based on the team cohesion conceptual model proposed by Carron in the context of sports, it is known that team cohesion can affect athletes’ personal behavior and attitudes (Carron, 1982). Considering the sub-dimensions of athlete engagement, which include confidence, dedication, vigor, and enthusiasm, existing research suggests that athletes’ identification with the team can transform into confidence (Fransen et al., 2016), and perceived team cohesion can positively affect athletes’ dedication and role engagement (Coleman et al., 2021). Thus, team cohesion can also influence athlete engagement by affecting its sub-dimensions. From the overall concept of athlete engagement, team cohesion represents the team’s attractiveness to individual members. The more cohesion athletes feel, the more likely they are to be attracted to the team, thereby being more willing to engage in training and competitions. Empirical research by Hodge et al. (2009), grounded in Self-Determination Theory, demonstrates that satisfying basic psychological needs is a critical antecedent factor influencing athlete engagement. In sports, cohesion is an important source of social support for athletes. When athletes receive more social support, they experience a higher sense of belonging and security, promoting the fulfillment of basic psychological needs and the stimulation of intrinsic motivation, leading to higher levels of athlete engagement. Furthermore, Chinese athletes, with their stronger collective sense, sense of responsibility, and interpersonal relationship needs, are more likely to project the purposes, tasks, and principles of group activities onto individual behavioral standards, and automatically adjust and adapt to the norms set by these benchmarks. Hence, Chinese athletes are more likely to perceive and convert team cohesion into their behaviors, such as being more engaged in training and competitions (Gu and Xue, 2022). Thus, the following hypothesis is proposed:

*H1*: Team cohesion has a positive impact on athlete engagement in collegiate basketball players.

### 2.2 The moderating role of paternalistic leadership by coaches

Paternalistic leadership by coaches refers to a style of leadership characterized by strict discipline and authority, paternalistic benevolence, and moral integrity within a personalized management atmosphere. This includes aspects of authoritarianism, benevolence, and moral leadership (Lin and Lian, 2016). The three-dimensional concept of paternalistic leadership suggests that the three types of leadership encompassed within it can be independently investigated, as proposed by teams (Fan and Zheng, 2000) and most subsequent researchers (Li and Wu, 2019). Thus, this paper considers the three leadership styles within paternalistic leadership as independent dimensions and explores their respective moderating roles. Human behavior is significantly influenced by the environment, and situational factors can significantly affect cognition and behavior, thereby moderating an individual’s attitudes and reactions when faced with stimuli (Shun and Wang, 2013). In sports teams, the team atmosphere perceived by athletes and the leadership style of coaches continuously influence athlete behavior. Team cohesion represents the influence of the team atmosphere on athletes, while the leadership style of coaches reflects the coaches’ impact on athletes. Under different coaching leadership styles, the effect of team cohesion on athlete engagement may vary.

Authoritarian leadership refers to a coach emphasizing that their authority is absolute and beyond challenge, exerting strict control over team members and demanding unconditional obedience from them (Lin and Lian, 2016). The theory of basic psychological needs, central to Self-Determination Theory (Zhang et al., 2011; Van Den Broeck et al., 2016), states that individuals have three basic psychological needs: autonomy, competence, and relatedness. The satisfaction of these needs can promote the internalization of extrinsic motivations, encouraging individuals to persist in activities longer, maintain a positive psychological state, grow more effectively, and produce more positive behavioral outcomes (Deci and Ryan, 2000). Environmental factors that hinder the satisfaction of these needs typically reduce individual initiative, job performance, and well-being (Reis et al., 2000; Liu and Zhang, 2010). When coaches employ authoritarian leadership, imposing strict control and demanding unconditional obedience, they may inadvertently reduce the athletes’ psychological need for autonomy, leading to resistance and decreased engagement. Under authoritarian leadership, negative emotions in athletes can emerge, potentially inhibiting the positive effects of team cohesion on athlete engagement. Furthermore, as the level of authoritarian leadership increases to a higher level, the positive impact of team cohesion on athlete engagement may not occur. Therefore, the following hypothesis is proposed:

*H2*: Authoritarian leadership negatively moderates the effect of team cohesion on athlete engagement in collegiate basketball players.

Benevolent leadership refers to a coach’s individualized, comprehensive, and sustained care for the well-being of their athletes (Lin and Lian, 2016). According to Social Exchange Theory, when team members receive recognition and care from their leaders, their work attitudes and emotional states significantly improve, gradually realizing their unique value within the team and, as a result, focusing more on their roles (Wu et al., 2016). In the sports context, the benevolent aspect of paternalistic leadership involves long-term and individualized care for subordinates. When athletes perceive this care, they may feel grateful to the coach and motivated to reciprocate. Additionally, the coach’s attention can help resolve conflicts privately and timely, fostering greater team unity. Therefore, when athletes experience benevolent leadership from their coach, they are more likely to translate team cohesion into increased engagement. Conversely, if the coach’s care is perceived as insufficient, the positive impact of team cohesion on athlete engagement may be limited. Thus, the following hypothesis is proposed:

*H3*: Benevolent leadership positively moderates the impact of team cohesion on athlete engagement in collegiate basketball players.

Moral leadership refers to a coach demonstrating high personal morals and professional competence, winning the admiration, emulation, and identification of the athletes (Lin and Lian, 2016). According to Social Learning Theory, human social behavior, attitudes, and values are influenced not only by direct experiences but also significantly by observational learning (Mo, 2002). In the sports context, where coaches and athletes interact closely, every action of the coach is observed by the athletes. When coaches display high moral standards and professional knowledge, athletes tend to admire and emulate these qualities, thereby fostering a fair and united team atmosphere. Therefore, when athletes perceive moral leadership from their coach, they are more likely to convert the sensed team cohesion into greater commitment to training and competitions. Conversely, if the level of moral leadership is lacking, team cohesion may not effectively enhance athlete engagement. Thus, the following hypothesis is proposed:

*H4*: Moral leadership positively moderates the impact of team cohesion on athlete engagement in collegiate basketball players.

**Figure 1:**
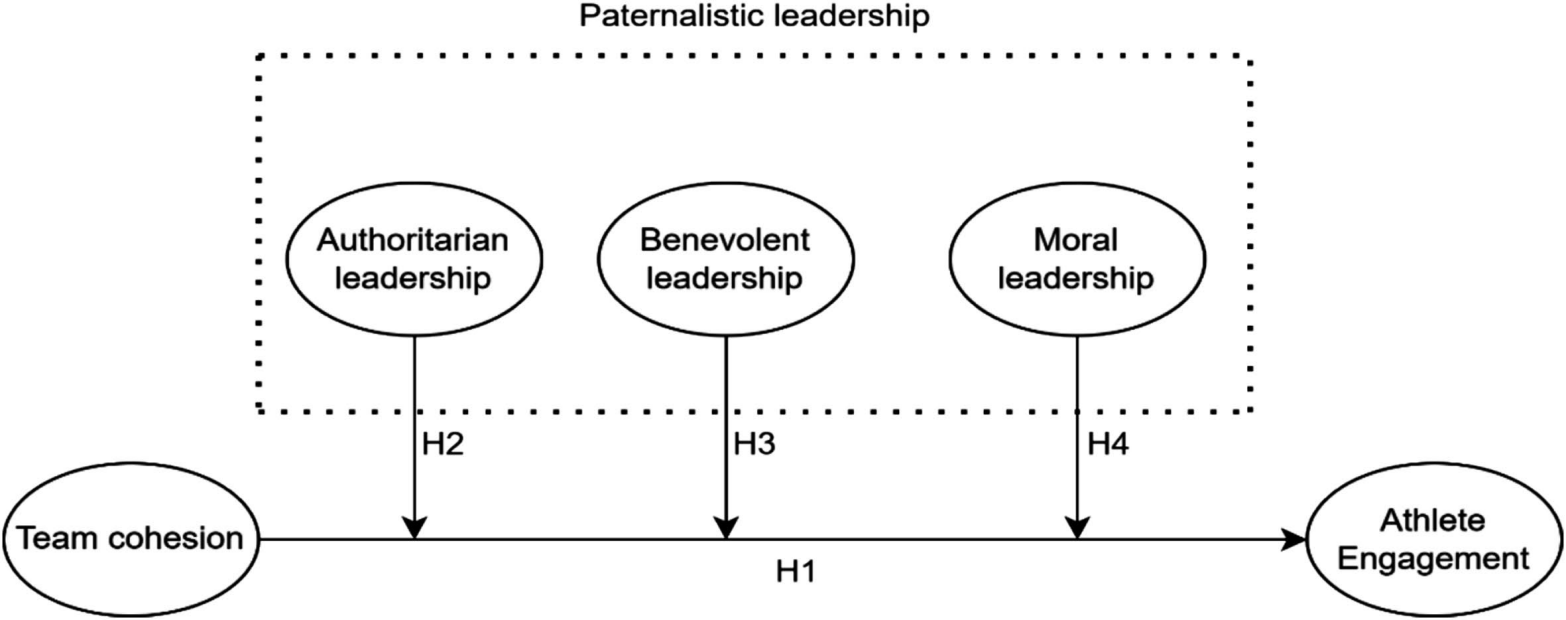
Conceptual model.

In summary, the hypothesized model to be tested in this paper is illustrated in Figure 1.

## 3 Research design

### 3.1 Research design and statistical analysis

This study was reviewed and approved by the Ethics Committee of Fujian Normal University. An anonymous survey was conducted through electronic links on the Wenjuanxing platform, with all participants voluntarily participating and signing electronic informed consent forms. In this study, Cronbach’s *α* coefficient in SPSS 26 was used to assess the reliability of the five scales, and structural equation modeling in AMOS 26 was employed to examine the discriminant validity among these scales. Additionally, descriptive statistics and correlation analyses were conducted in SPSS 26 to present the current status of the variables and their interrelationships. Interaction terms were generated by multiplying the mean score of team cohesion with the mean scores of authoritarian leadership, benevolent leadership, and moral leadership, respectively, to create three moderating variables. Hierarchical regression analysis in SPSS 26 was then utilized to examine the direct effect of team cohesion on athlete engagement as well as the moderating effect of paternalistic leadership. To further clarify the range of the moderating effects of paternalistic leadership, simple slope tests and the Johnson-Neyman technique in Hayes’ PROCESS macro (Hayes, 2013) were applied, and visualizations were created in Excel to more precisely define and present the range of moderating effects.

### 3.2 Study sample

Convenience sampling was utilized to select athletes participating in the 26th China Collegiate Basketball League from provinces and cities including Beijing, Liaoning, Shandong, Anhui, Fujian, Hunan, Hubei, Guangdong, and the Ningxia Hui Autonomous Region. A total of 404 questionnaires were collected, and after excluding those that were invalid due to patterned responses, 361 valid questionnaires were obtained. The sample included 234 males and 127 females, with an average age of 20.40 years (standard deviation = 2.00). The participants were categorized as follows: 198 athletes from the first-tier league, 119 from the second-tier, and 44 from the third-tier; 87 were national-level athletes or higher, 120 were second-tier national athletes, and 154 had no athletic rank.

### 3.3 Variable measurement

The scales used in this study are well-established within the country and have been employed in at least three high-level academic articles. Team cohesion was measured as the independent variable using the Group Environment Questionnaire translated and revised by Ma (2004), which consists of 15 items across four dimensions: group task attraction, group social attraction, group task consistency, and group social consistency, with 3, 4, 4, and 4 items, respectively. A 7-point Likert scale ranging from “strongly disagree” (1) to “strongly agree” (7) was used. The Cronbach’s *α* coefficient for this scale in the study was 0.928.

Athlete engagement was measured as the dependent variable using the Chinese version of the Athlete Engagement Questionnaire translated and revised by Ye (2014), which includes 16 items across four dimensions: confidence, vigor, dedication, and enthusiasm, with 4 items each. A 5-point Likert scale ranging from “never” (1) to “always” (5) was employed. The Cronbach’s α coefficient for this scale in the study was 0.963.

Three moderating variables—authoritarian leadership, benevolent leadership, and moral leadership of coaches—were measured using the Paternalistic Leadership Scale for Sports Coaches revised by Gao et al. (2013). This scale uses a 6-point Likert scale from “strongly disagree” (1) to “strongly agree” (7) and contains 15 items across three dimensions. Specifically, authoritarian leadership includes 5 items with a Cronbach’s *α* coefficient of 0.882; benevolent leadership has 4 items with a Cronbach’s *α* coefficient of 0.920; and moral leadership has 4 items with a Cronbach’s *α* coefficient of 0.950.

## 4 Research results

### 4.1 Test for common method bias

In this study, the Harman single-factor test was employed to check for common method bias. All items from the five constructs in the questionnaire were analyzed together in a factor analysis. Without rotation, the first principal component accounted for 39.342% of the variance, which is less than 50%. This indicates that although the survey subjects, measurement methods, and timing of measurement were the same, there was no significant common method bias present in this study (Podsakoff and Organ, 1986).

### 4.2 Confirmatory factor analysis

Confirmatory factor analysis of the five constructs—team cohesion, athlete engagement, authoritarian leadership, benevolent leadership, and moral leadership—was conducted using Amos 26.0 (see Table 1). The results showed that the five-factor measurement model had the best fit (*χ*^2^ = 421.556, df = 179, *χ*^2^/df = 2.355, CFI = 0.966, NFI = 0.942, RMSEA = 0.061) and was significantly better than the four-factor and other models, indicating good discriminant validity among the five constructs in this study.

**Table 1:** Multilevel confirmatory factor analysis.

Model	*χ* ^2^	df	*χ*^2^/df	CFI	NFI	RMSEA
Five-factor model: team cohesion, athlete engagement, authoritarian leadership, benevolent leadership, moral leadership	421.556	179	2.355	0.966	0.942	0.061
Four-factor model: team cohesion, athlete engagement, authoritarian leadership, combined benevolent and moral leadership	1660.397	183	9.073	0.792	0.773	0.15
Three-factor model: team cohesion, athlete engagement, combined authoritarian, benevolent, and moral leadership	3302.709	186	17.756	0.561	0.548	0.216
Two-factor model: team cohesion, combined athlete engagement, authoritarian, benevolent, and moral leadership	3691.633	188	19.636	0.507	0.495	0.228
Single-factor model: combined team cohesion, athlete engagement, authoritarian, benevolent, and moral leadership	4242.821	189	22.449	0.429	0.42	0.244

### 4.3 Correlation analysis

Spearman’s correlation analysis was used to test the relationships among the five variables: team cohesion, athlete engagement, authoritarian leadership, benevolent leadership, and moral leadership. The means, standard deviations, and correlation coefficients of these variables are shown in Table 2. There were significant positive correlations between team cohesion, athlete engagement, benevolent leadership, and moral leadership. Authoritarian leadership was significantly negatively correlated with team cohesion, athlete engagement, and moral leadership, but it showed no significant correlation with benevolent leadership.

**Table 2:** Descriptive statistics and correlational analysis of variables.

Variables	*M*	SD	1	2	3	4	5
1. Team cohesion	6.196	0.78	1				
2. Athlete engagement	4.225	0.652	0.572^***^	1			
3. Authoritarian leadership	3.071	1.288	−0.325^***^	−0.184^***^	1		
4. Benevolent leadership	4.334	1.303	0.328^***^	0.400^***^	0.032	1	
5. Moral leadership	5.123	1.035	0.576^***^	0.559^***^	−0.144^**^	0.487^***^	1

*n* = 361, *p < 0.05, **p < 0.01, ***p < 0.001.

### 4.4 Hierarchical regression analysis

This study employed hierarchical regression analysis to test direct effects and moderating effects. Table 3 presents the regression analysis process and results. The hierarchical regression model was structured as follows: the first layer included three control variables (gender, age, league level, and athlete ranking); the second layer included the independent variable (team cohesion); the third layer included the three moderating variables (authoritarian leadership, benevolent leadership, and moral leadership); and the fourth layer included the interaction terms between the three moderating variables and the independent variable (the interactions of authoritarian leadership, benevolent leadership, and moral leadership with team cohesion, respectively). The results indicated (see Table 3) that after controlling for demographic variables, Model 2 showed that team cohesion had a significant positive impact on athlete engagement (*B* = 0.442, *p* < 0.001), confirming Hypothesis 1. As indicated by Model 4, the model fit significantly improved after adding the interaction terms (Δ*R*^2^ = 0.021, *p* < 0.05). Specifically, the interaction term between team cohesion and authoritarian leadership has a significant negative effect on athlete engagement (*B* = −0.072, *p* < 0.05), confirming Hypothesis 2. The interaction between team cohesion and benevolent leadership did not significantly affect athlete engagement (*B* = −0.043, *p* > 0.05), thus Hypothesis 3 was not supported. The interaction between team cohesion and moral leadership had a significant positive impact on athlete engagement (*B* = 0.071, *p* < 0.05), supporting Hypothesis 4.

**Table 3:** Hierarchical regression analysis among variables.

	Variables	Model 1	Model 2	Model 3	Model 4
Control variables	(Constants)	4.821^***^	1.701^***^	1.426^**^	0.851
	Gender	−0.301^***^	−0.111	−0.06	−0.066
	Age	−0.004	−0.004	−0.007	−0.003
	Competitive level	0.046	0.033	0.096	0.084
	Athlete rank	−0.115	−0.015	−0.006	−0.015
Independent variable	Team cohesion		0.442^***^	0.263^***^	0.328
Moderating variables	Authoritarian leadership			0.001	0.446^*^
	Benevolent leadership			0.086^*^	0.347
	Moral leadership			0.174^***^	−0.25
Interaction terms	Team cohesion × Authoritarian leadership				−0.072^*^
	Team cohesion × Benevolent leadership				−0.043
	Team cohesion × Moral leadership				0.071^*^
*R* ^2^		0.107	0.337	0.436	0.457
Adjusted *R*^2^		0.097	0.327	0.424	0.44
Δ*R*^2^		0.107^***^	0.230^***^	0.100^***^	0.021^*^
*F*		10.642^***^	122.943^***^	20.773^***^	4.516^*^

*p < 0.05, **p < 0.01, ***p < 0.001.

**Figure 2:**
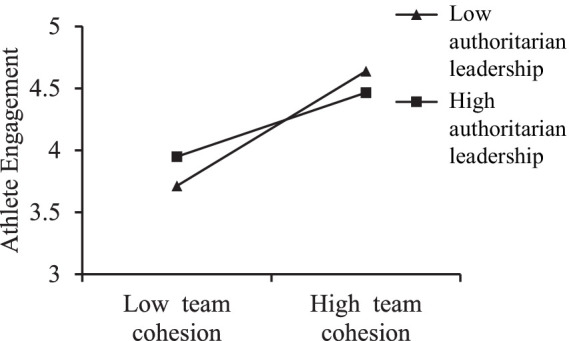
The moderating role of authoritarian leadership in the relationship between team cohesion and athlete engagement.

**Figure 3:**
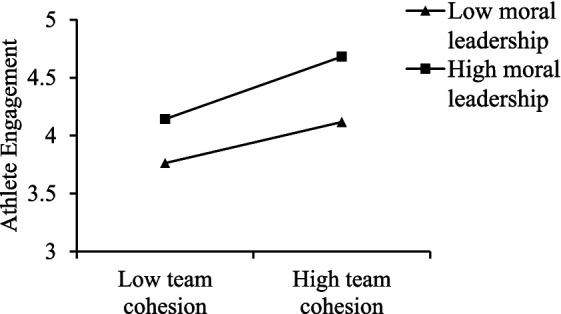
The moderating role of moral leadership in the relationship between team cohesion and athlete engagement.

To more clearly reveal the moderating effects of authoritarian and moral leadership on the relationship between team cohesion and athlete engagement, simple slope tests were conducted using data obtained from the SPSS macro PROCESS developed by Hayes (2018). Following the recommendation of Aiken (1991), a simple slope test was conducted using the mean ± 1 standard deviation (SD) as indicators of high and low levels. Four points were plotted to illustrate the impact of high and low team cohesion on athlete engagement under conditions of high and low authoritarian leadership and high and low moral leadership. These points were then connected with lines for visualization, thereby demonstrating the moderating effects of different leadership styles. For authoritarian leadership, as shown in Figure 2, at a low level of authoritarian leadership (−1SD), team cohesion had a stronger impact on athlete engagement (*B* = 0.625, *t* = 10.790, *p* < 0.001); at a high level of authoritarian leadership (+1SD), the impact was weaker (*B* = 0.367, *t* = 7.250, *p* < 0.001). For moral leadership, as shown in Figure 3, at a low level of moral leadership (−1SD), team cohesion had a weaker impact on athlete engagement (*B* = 0.227, *t* = 4.688, *p* < 0.001); at a high level of moral leadership (+1SD), the impact was stronger (*B* = 0.346, *t* = 5.995, *p* < 0.001).

**Figure 4:**
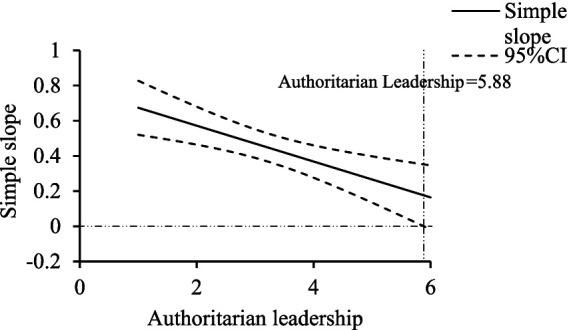
Simple slopes of authoritarian leadership’s moderating effect.

**Figure 5:**
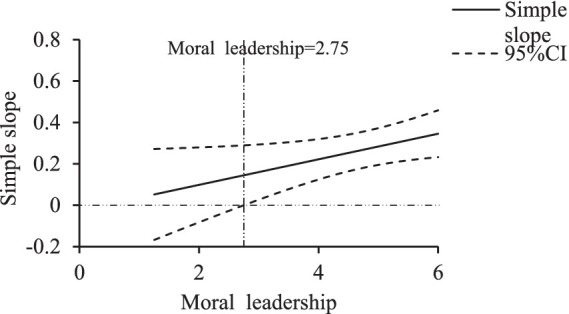
Simple slopes of moral leadership’s moderating effect.

This study also used the Johnson–Neyman technique within the SPSS macro PROCESS developed by Hayes (2013) to explore the intervals where authoritarian and moral leadership have significant moderating effects. For authoritarian leadership, as shown in Figure 4, when the authoritarian leadership score ranged between 1 and 5.88, the simple slope’s 95% Bootstrap confidence interval (CI) did not include zero, indicating a significant effect of team cohesion on athlete engagement, with the effect decreasing as authoritarian leadership increased, until it was no longer significant when the authoritarian leadership score exceeded 5.88. For moral leadership, as shown in Figure 5, when the moral leadership score ranged between 2.75 and 6, the simple slope’s 95% Bootstrap CI did not include zero, indicating a significant effect, with the effect increasing as moral leadership increased, and becoming non-significant when the moral leadership score was below 2.75.

## 5 Discussion

With the advancement of positive psychology, athlete engagement, as the counterpart to burnout, has garnered significant attention from scholars, making the exploration of strategies to enhance athlete engagement a critical research topic. This study approached from an organizational situational perspective, exploring the impact of team cohesion on the engagement of collegiate basketball athletes and investigating whether the three types of leadership within paternalistic leadership have a moderating effect on the relationship between team cohesion and athlete engagement, as well as the nature of these effects. The results showed that team cohesion has a positive impact on athlete engagement, and that moral and authoritarian leadership within paternalistic leadership play moderating roles.

### 5.1 Direct impact of team cohesion on athlete engagement in collegiate basketball players

The findings regarding team cohesion and athlete engagement corroborate parts of Social Information Processing Theory, indicating that environmental factors directly affect athletes’ attitudes and behaviors. The results also support the sports group cohesion model proposed by the scholar Carron, showing that team cohesion impacts individual outcomes for athletes. From an empirical research perspective, the findings of this study are consistent with previous research (Gu and Xue, 2022; Ivaskovic and Cater, 2022), which found that team cohesion has a significant positive impact on athlete engagement. Unlike previous studies that focused on professional athletes from various sports, this study focused on collegiate basketball athletes, demonstrating that team cohesion positively affects both professional and collegiate athletes, thereby increasing the generalizability of the empirical conclusions about their relationship. This might be because Social Information Processing Theory points out that team environmental factors constantly impact athletes, with cohesion being an important component of these factors. According to Self-Determination Theory, the satisfaction of basic psychological needs can enhance athlete engagement (Hodge et al., 2009); the perception of team cohesion can provide athletes with a sense of belonging, satisfying their basic psychological needs and thus affecting their level of engagement. This study surveyed Chinese basketball athletes, who possess a strong collective spirit and a high sense of organizational identification, preferring a united team atmosphere. The impact of team cohesion on athletes is more pronounced and more easily translates into their behavioral attitudes. This all underscores the pivotal role of team cohesion in shaping athletes’ attitudes and behaviors, highlighting that enhancing team cohesion is an effective way to boost athlete engagement in collegiate basketball players.

Furthermore, some scholars have focused on the impact of engagement on team cohesion (Ma and Wang, 2006; Zhang, 2007), rather than the impact of team cohesion on athlete engagement. Both types of research are significant but from different perspectives. These scholars are interested in how individual actions can enhance the team atmosphere, whereas this paper focuses on how organizational situational factors influence individual behaviors. According to the broaden-and-build theory of positive emotions, positive emotions can enhance physical, intellectual, interpersonal, and psychological resources, improving athletes’ abilities and adaptability, and thereby improving the quality of relationships among team members (Guo and Wang, 2007). This in turn enhances team cohesion, which brings social support and a sense of belonging to the athletes, thus promoting their level of engagement. Hence, it is evident that these factors can influence each other.

### 5.2 The moderating role of paternalistic leadership

The leadership style of a leader is an important organizational situational factor that continuously impacts the team. Paternalistic leadership, a prevalent style among Chinese leaders, has received considerable attention from scholars. In the field of business management, researchers have found that paternalistic leadership has a moderating effect between team atmosphere and team member behavior (Shi et al., 2018; Li and Wu, 2019). However, in the sports domain, most studies on coaches’ paternalistic leadership have focused on its direct effects on athlete behavior or team atmosphere. Few scholars have examined the moderating role of coaches’ paternalistic leadership styles on the relationship between team atmosphere and team member behavior.

In terms of authoritarian leadership, this style negatively moderates the relationship between team cohesion and athlete engagement in collegiate basketball players. Specifically, when collegiate basketball players experience authoritarian leadership within the range of [1, 5.88], it suppresses the impact of team cohesion on the engagement of collegiate basketball players. The stronger the perceived authoritarian leadership, the weaker the impact of team cohesion on athlete engagement, until the authoritarian leadership score exceeds 5.88, beyond which team cohesion no longer significantly affects athlete engagement. As collegiate students mature in their self-awareness, they exhibit a strong tendency toward independence and a desire for self-selection, both in life and in training and competitions, seeking greater autonomy in decision-making. Additionally, collegiate students typically have higher self-esteem, demand respect from others, and are sensitive and likely to show strong emotional reactions to actions that infringe on their self-esteem (Xiong et al., 2015). For such athletes, when coaches display an authoritarian leadership style by strictly controlling athletes, harshly criticizing them, and demeaning their abilities and contributions, it reduces the athletes’ sense of psychological need fulfillment and psychological well-being (Du and Liu, 2016), dampening their enthusiasm and confidence for training and competition, and may even lead to rebellious attitudes, creating an oppressive atmosphere. This can negate the positive effects of team cohesion on athlete engagement. When the score for authoritarian leadership exceeds 5.88, athletes frequently feel controlled by the coach, and at this point, the role of team cohesion in enhancing athlete engagement in collegiate basketball players becomes less apparent.

However, in recent years, many scholars have called for a reassessment of the role of authoritarian leadership. Zhou et al. (2010) have categorized authoritarian leadership into two types based on different control focuses: discipline-focus authoritarian leadership, which emphasizes leaders strictly monitoring subordinates’ tasks and work processes to achieve high performance, and dominance-focus authoritarian leadership, which emphasizes the personal authority of the leader and control over subordinates (Wang et al., 2023). Studies have found that discipline-focus authoritarian leadership by sports coaches can enhance athletes’ positive behaviors and psychological energy, while dominance-focus authoritarian leadership tends to negatively affect athletes’ effortful behavior and psychological energy (Zheng et al., 2019; Pan W. et al., 2022). Moreover, when coaches adopt discipline-focus authoritarian leadership while providing support to athletes, they are more likely to nurture athletes with positive emotions and behaviors (Xu et al., 2023). Therefore, discipline-focus authoritarian leadership could positively modulate the relationship between team cohesion and athlete engagement. The scales used in this study lean more toward discipline-focus authoritarian leadership, hence the observed negative moderating effect. Thus, when collegiate basketball coaches interact with collegiate athletes, they should maintain an appropriate level of strictness in training but avoid overly harsh criticism and control, prevent the team atmosphere from becoming too oppressive, and grant athletes appropriate autonomy in decision-making to foster good team cohesion and enhance athlete engagement.

In terms of benevolent leadership, this study did not find it to have a moderating effect between team cohesion and athlete engagement in collegiate basketball players, which could be due to two main reasons. Firstly, based on the measurement items and conceptual definitions, benevolent leadership involves not only care during training and competitions but also concern for athletes’ families and personal lives. Scholars have also found that benevolent leadership, which often pertains more to subordinates’ personal lives, does not significantly affect organizational citizenship behaviors, role behaviors (Li, 2021), or athlete satisfaction (Liu et al., 2022). Unlike professional athletes and sports school students, collegiate basketball players are adults and their lives are not solely defined by training and competition. They maintain a clear boundary between life and basketball and sometimes do not welcome coaches’ excessive interference in their non-sporting lives. Secondly, benevolent leadership emphasizes a coach’s personal care for athletes, and while it might directly impact athletes or the team, the personal care perceived by athletes might not moderate the influence of team factors on individual outcomes. It is important to note that even though benevolent leadership did not show a significant moderating effect in the relationship between team cohesion and athlete engagement in collegiate basketball players, collegiate basketball coaches should not neglect care and support for their athletes, as most studies conclude that benevolent leadership directly has a positive impact on both athletes and teams.

In terms of moral leadership, this approach plays a positive moderating role between team cohesion and athlete engagement in collegiate basketball players. Specifically, when athletes perceive moral leadership in the range of [2.75, 6], it significantly enhances the effect of team cohesion on the engagement levels of collegiate basketball players. Furthermore, the stronger the perceived moral leadership, the greater the impact of team cohesion on athlete engagement. Confucian culture, as a core component of Chinese traditional culture, profoundly influences the Chinese national character, moral standards, and value orientations, serving as a subtle moral guide in daily decision-making (Pan Z. et al., 2022). In Confucian thought, great importance is placed on the personal virtue of leaders, believing that the virtue of a nation’s ruler is fundamental to the state’s prosperity. For instance, it is written in the “Analects of Confucius, The Practice of Government” that “governance through virtue can make a leader the North Star, around which all other stars revolve,” suggesting that leaders, through moral governance, can become focal points and unifying cores of their groups (Hu and Li, 2022).

A leader’s noble virtues displayed before the public can serve as a role model for behavior, influencing not just external actions but also fostering integrity and sincerity in beliefs and attitudes through subtle influences. Over time, this socialization process has led people to gradually transform Confucian emphasis on leaders’ virtues into a societal consensus. Even in today’s society and organizations, the vast majority of Chinese people expect leaders to demonstrate high personal morals (Wang, 2014). A coach, as the direct leader of a team, when endowed with both character and competence, can make athletes feel a greater sense of fairness in the team (Yu et al., 2018), and more willing to believe that a Moral Coach can lead them to success. Athletes emotionally identify more with their team (Tang and Song, 2004), enhancing the positive impact of team cohesion on athlete engagement. Conversely, if a coach lacks convincing abilities and does not act as a role model or treat everyone fairly, even with strong team cohesion, team members may feel uneasy, distrust the coach’s decisions, and find it difficult to fully commit to training and competitions. When the moral leadership score is below 2.75, meaning collegiate basketball players perceive the collegiate basketball coach’s virtues and competence as weak, the impact of team cohesion on athlete engagement in collegiate basketball players becomes insignificant.

Coaches should continuously learn and lifelong improve their knowledge and skills to earn the respect and trust of athletes. Moreover, it is crucial for coaches to maintain high moral standards. With the development of social media, collegiate students have easy access to knowledge, which they can acquire without the intermediary of a coach, making a coach’s expertise less critical than before. The virtues displayed by the coach become a vital factor in convincing athletes and morally educating them. Collegiate basketball coaches need to lead by example, treat every athlete fairly, and avoid favoritism to maintain a positive team atmosphere.

## 6 Summary

Firstly, team cohesion has a significant positive impact on athlete engagement in collegiate basketball players.

Secondly, authoritarian leadership significantly negatively moderates the relationship between team cohesion and athlete engagement in collegiate basketball players, with stronger authoritarian leadership weakening the moderating effect. When the authoritarian leadership score exceeds 5.88, team cohesion no longer significantly affects athlete engagement in collegiate basketball players.

Thirdly, benevolent leadership does not have a moderating effect between team cohesion and athlete engagement in collegiate basketball players.

Fourthly, moral leadership significantly positively moderates the relationship between team cohesion and athlete engagement, with higher moral leadership strengthening the moderating effect. When the moral leadership score is below 2.75, team cohesion no longer significantly impacts athlete engagement in collegiate basketball players.

## 7 Limitations and future outlook

First, this study focuses on athletes in the China Collegiate Basketball League to explore the moderating role of paternalistic leadership in the relationship between team cohesion and engagement among collegiate basketball players. The sample is limited to a single sport and specific competitive level, which may not fully represent athletes from other sports or competitive tiers. Future research could expand to different sports and various competitive levels, enabling comparative analysis of the applicability of team cohesion and leadership styles, thereby providing broader theoretical support for coaching strategies across diverse contexts.

Secondly, this study utilized a cross-sectional quantitative design, which limits its capacity for causal inference. Future research could employ longitudinal surveys or experimental designs to enhance the robustness of causal conclusions.

Third, for the purposes of this study, we focused exclusively on the moderating effects of authoritarian, benevolent, and moral leadership within the framework of paternalistic leadership. However, coaching leadership styles are varied, and both authoritarian leadership (Zhou et al., 2010) and benevolent leadership (Lin et al., 2022) have recently been further subdivided into more nuanced categories. Future research could integrate additional leadership styles or conduct an in-depth investigation into specific types within paternalistic leadership to provide a more comprehensive understanding of the coach-athlete relationship and its impacts.

## Data Availability

The raw data supporting the conclusions of this article will be made available by the authors, without undue reservation.
